# 

**Published:** 1887-08-06

**Authors:** 


The Hospital.

**An Institution, Family, and Parochial Journal of Hospitals, Asylums, and all Agencies for the Care of the Sick, Criticism and News.**

SATURDAY, AUGUST 6TH, 1887.

### Notice.

On and after August 1st, 1887, The Hospital will be published at 2, Paternoster Square, E.C. Mr. A. P. Watt will, from the date named, assume the business management of the Journal, and all communications relative to the Publishing and Advertising Departments should, after August 1st, be sent to him. All accounts due for advertising in The Hospital from April 1st, 1887, must be paid to Mr. A. P. Watt, whose receipt will be sufficient discharge.

### NOTICE TO CORRESPONDENTS.

All correspondence must be written on one side of the paper only. We cannot undertake to return MSS. not used.

**Communications**, whether intended for insertion or for private information, must be authenticated by the names and addresses of the writers, not necessarily for publication.

**Local papers** containing reports or news paragraphs **should be marked.**

It is especially requested that early intelligence of **local events of interest**, or which it may be thought desirable to bring under notice, may be sent direct to the Editor.

Communications respecting editorial matters should be addressed to the Editor, Norfolk House, Norfolk Street, Strand, London, W.C.

### The Literary Prize.

A prize of **Two Guineas** will be given every month for the best story or incident based upon hospital, or asylum, or student life, or any incident connected with the professions of medicine or nursing.

### QUESTIONS AND ANSWERS.

All questions should be addressed to Mr. Howard J. Collins, Secretary, The Hospitals Association, Norfolk House, Norfolk Street, Strand, W.C.

### MAY A YOUNG LADY BE DETAINED IN AN ASYLUM AGAINST HER PARENTS' WISHES?

"Inquirer" does not give sufficiently detailed and precise particulars to enable us to answer this question in her case. Will she state fully the circumstances which induced the two magistrates to place the young lady (aged 32) in a county asylum as a pauper, upon information given by a relative, without the consent of the parents, who offered to provide "their medical attendant, a hospital nurse, and every comfort in their own home"? There must surely have been a very strong case presented before the magistrates made such an order. Upon information given to the Relieving Officer, or Overseer, or constable, he is bound to take steps in the matter, and if the alleged lunatic be "not under proper care and control," or "cruelly neglected or ill-treated," or "wandering at large," he can be removed to an asylum on the order of two magistrates, a "medical certificate" and a "statement" signed by one or other of the above-named officials. The patient would be paid for either by the county, or borough, or Board of Guardians, and these would recoup themselves by making an order on the relatives of the patient, provided the relatives were able to pay. The above are the terms of the Act, and no one can legally go beyond it. The Visiting Justices (any three of them) have power to discharge any patient, and almost invariably do so, upon application of the relatives, unless the Medical Superintendent is prepared to state that the patient is homicidal or suicidal. The relatives should apply under the 16 and 17 Vict., chap. 97, sect. 81. This failing, let them take legal advice. Of course the patient could be made a private patient.—[Ed. T. H.]

### The Children's Corner.

**Fourth Quarterly Prize Competition.**
Began 2nd July, 1887 ; ends 24th Sept., 1887.

**Puzzles—Sixth Week.**

No. 22. A Bouquet of Flowers. 1. Frozen water, and a particle of it melted. 2. An animal and a girl's name. 3. A colour and something found in a church. 4. A wise man's impression.

No. 23. Double Acrostic. The initials read downwards give a favourite game, and the finals read upwards. give the name of a person who takes part in it. 1. Part of a ship. 2. A country of Europe. 3. A city of Asia Minor. 4. A seaport of France. 5. A county of England. 6. A puzzle. 7. A wooden vessel.

No. 24. Historical Charade. My first is the opposite to cold ; my second a stimulus to action ; my whole a hero in history. Tell me his name and where he died.

No. 25. Square Word. 1. Part of a bird. 2. Thought. 3. Not untidy. 4. An opening into a field.

**Letters—Sixth Week.**

Next week write me an account of your favourite *Fruits*, when they are in season, and where they are to be found.

**Results of Fourth Week—Puzzles.**

No. 13. Ornithological Charade. The shy and timid bird is the Kingfisher.

No. 14. No one has guessed the answer to the conundrum this week. It is rather a difficult one, but very good, as I think you will all agree. *Ans.* The sun takes away its character, and the moon casts reflections upon it.

No. 15. Diamond Puzzle.

S

s I n c o S y

S I S T E R S

c h E a t i R e

S

No. 16. Anagrams. The rivers are the Rhone, Garonne, Medway, and Boyne.

*All right*—None ; *Three right*—Toby, Scrooge, A. G. S. McCulloch, Mount Edgcumbe, The Eda, Ethel, Amy, Alsie, Joe, Agatha, A. C. K., Umslopogaas ; *Two right*—Poodle, Mikado, Le Cerf Agile, Ossian, Skye, Princess Ida ; *One right*—Ellie, Socrates.

**Letters.**

The letter-subject for this week is pronounced by universal consent to be a favourite one. Toby and Socrates have again sent us very good letters, which we are unable to insert, as theirs appeared last week.

Picnics, Scotch and Otherwise.

Here is Skye's (aged 14) account of a Scotch picnic, and a very delightful *al fresco* gathering it must be :—The picnics that I have been to in England have not been many, but I enjoyed them very much, always because they were in the country ; and food taken in the open air, and in an irregular way, also seems so much better than at home. Then the ramble and scramble after flowers or anything is so delightful. In Scotland picnics are arranged by a few jolly people, who send invitations to others, adding, Any contributions to the above will be received by Mrs. So-and-so. The evening preceding the picnic each person invited contributes some comestibles. The gentlemen provide the carriage, horses and champagne, the ladies do all the rest. These picnics go a distance of thirty miles to the foot of some mountain, and near a lovely loch. In the evening, before starting for home, they form a circle, and crossing their neighbours' hands, sing " Auld lang syne" with a hearty goodwill. These picnics are never forgotten, not because there was a red currant tart, but for the " trusty freends" that were there.

Le Cerf Agile (aged 13) writes from Leicester:—We once went to a picnic at the Hanging Rocks. It was great fun to spread the cloth on the smoothest place we could find ; we had to put a stone at each corner to keep it down. Some of us cut up the cucumbers, and others laid the knives and forks, and one little girl came to grief in carrying a red-currant tart ; her foot slipped, and down she came, and her nice white frock was all stained with the juice. After dinner we roamed about gathering ferns and wildflowers, and some of us got lost in a wood, and were rather frightened when we came upon a gipsy tent ; the woman was nursing such a pretty, dark-skinned little baby, and there was a big gipsy boy who showed us our way out of the wood. We could not understand all he said, but he seemed very pleased with the pennies we gave him. Then we all went to a farm-house for new milk and bread and butter, and the people let us go into the shed to see the cows milked. There was one Alderney, such a quiet, gentle creature, and a beautiful little Brittany cow, hardly bigger than a St. Bernard dog. It was a very happy day altogether, and I think picnics are very nice.

Ethel relates how at a picnic " a cow ate up the bread and butter and cake, and upset six cups of tea and a jug of hot water over its foot !" Ossian delights to take his dinner to Cobham Woods, and there to play at robbers. And in reply to my suggestion that a cow and a red-currant tart might be introduced on the scene, adds : " I have never had a red-currant tart nor a cow at a picnic, so I cannot say how they tasted." Amy gives an account of a visit to the caves in the Castle grounds at Reigate, where she went into the Guards' room, and heard a man sound the gong, said to have been used by the Barons. Here is a letter from Ellie, aged 12 :—

It is a good many years ago, but my story will still hold good. A party of lords and ladies assembled together in a beautiful forest in the North Riding of Yorkshire, having previously resolved to have a picnic there ; and a jolly time they had of it, I know, from what I was told (this is a true story, Mr. Editor), for after the picnic they joined in the sport of archery, the ladies shooting about a hundred paces in advance of the gentlemen, to be nearer the target ; and whilst they were thus engaged, a party of schoolboys from a neighbouring boarding school, having been rambling through the forest, bent on all sorts of mischief, suddenly espied the remains of the repast, so down they all sat, took French leave, and helped themselves. Of all the good things that were there I don't recollect the red-currant tart, but I know there was some blanc-mange, and plenty of it—at least one of the boys that was there told me so.

Three marks each :—Toby, Socrates, Skye, Ellie, Le Cerf Agile. Two marks each :—Amy, Mikado, Ethel, Mount Edgcumbe, Ossian.

**Notices to Correspondents.**

Mikado and Le Cerf Agile.—I think you will quite understand why I have not credited your answer to No. 14. Thanks for the puzzle.

N.B.—All letters must reach the " Puzzle Editor" not later than the first post on Thursday.

### Amusements with Profit.

### PRIZE COMPETITIONS.

**Quarterly Prize Competition.**
Began 23rd July ; ends 15th October.

**A Prize of Three Guineas**

will be awarded to the Competitor who shall have sent in the largest number of correct or best answers. No one will be permitted to win the Three Guinea Prize twice in any one year, but we shall award annually an additional

**Prize of Five Guineas**

to the competitor who shall have sent in the largest number of correct or best answers during the twelve months.

**No. 5. Double Acrostic.**

Two men of learning and of wit,
Beneath whose feet all poets sit ;
Their age long centuries divide,
Yet bookshelves find them side by side.

My *first* goes begging through the land,
Through village, town, or seaport strand ;
My *second* tells you what you are ;
My *third* falls from Apollo's car ;
My *fourth* makes stubborn cattle go ;
My *fifth* indignant monarchs show ;
My *sixth* and last rhymes well with nation,
And does not mean incarceration.

**No. 6. Rhythmical Recreation.**

The Healing Art.

The following spaces are to be filled up poetically.

| | |
|---|---|
| When | pain, |
| Crowds | new, |
| How | again, |
| Which | view. |
| All | art, |
| Which | smart |

**Answers.**

No. 1. Double Acrostic.

| | | |
|---|---|---|
| T | hunde | R |
| A | jacci | O |
| M | o | B |
| O | racl | E |
| S | ta | R |
| H | orne | T |
| A | nolle | B |
| N | autil | U |
| T | eache | R |
| E | de | N |
| R | omulu | S |

Correct answers have been received from K. M., Mrs. V. Wilson, Ellice, Mater, Gretta, H. B., Perseverance, Condorcet, Esau, Boss, Aralc, Common Sense, Lex, Ostrich, Brownie, Tabs, Man of the Sea.

No. 2. Enigma.—*Ans.* An Army.

Correct answers have been received from Mrs. V. Wilson, Ellice, Mater, Perseverance, Ostrich, Condorcet, Esau, Boss, Aralc, Common Sense, H. B., Tabs, Lex, Man of the Sea, Brownie.

